# Loss of aquaporin-4 impairs cerebrospinal fluid solute clearance through cerebrospinal fluid drainage pathways

**DOI:** 10.1038/s41598-024-79147-y

**Published:** 2024-11-14

**Authors:** Daisuke Kato, Hiroyuki Kameda, Naoya Kinota, Takaaki Fujii, Bai Xiawei, Zhou Simi, Yoshiki Takai, Simon Chau, Yoshiki Miyasaka, Tomoji Mashimo, Yoichiro Abe, Masato Yasui, Kazuyuki Minowa, Kohsuke Kudo

**Affiliations:** 1 Department of Diagnostic and Interventional Radiology, Hokkaido University Hospital, Sapporo, Japan; 2 Department of Diagnostic Imaging, Graduate School of Medicine, Hokkaido University, Sapporo, Japan; 3 Department of Radiology, Faculty of Dental Medicine, Hokkaido University, Sapporo, Japan; 4 Department of Neurology, Tohoku University Graduate School of Medicine, Sendai, Japan; 5 Department of Pathology, Tohoku University Hospital, Sendai, Japan; 6 Department of Pharmacology, Keio University School of Medicine, Tokyo, Japan; 7 Laboratory of Reproductive Engineering, Institute of Experimental Animal Sciences, Osaka University Medical School, Suita, Japan; 8 Division of Animal Genetics, Laboratory Animal Research Center, Institute of Medical Science, The University of Tokyo, Tokyo, Japan

**Keywords:** Aquaporin-4, Glymphatic System, Neurofluid, Dynamic contrast-enhanced MRI, Neurology, Preclinical research, Neuronal physiology

## Abstract

The aquaporin-4 (AQP4) water channel is essential in neurofluid dynamics. AQP4 loss impairs solute exchange between the cerebrospinal fluid (CSF) and interstitial fluid (ISF). However, whether AQP4 expression affects solute clearance from the CSF space to the extracranial space remains unclear. This study aimed to investigate this using dynamic contrast-enhanced magnetic resonance imaging (DCE-MRI) following the intrathecal administration of gadolinium-based contrast agents (GBCAs) to AQP4 knockout (KO) rats. AQP4 KO rats showed reduced efflux of intrathecal GBCAs to the extracranial spaces through CSF drainage pathways and increased retention of intrathecal GBCAs in the CSF space compared with the controls. These results suggest that AQP4 loss impairs solute clearance from the CSF space to the extracranial spaces via the CSF drainage pathways. This study revealed a close relationship between AQP4 expression and CSF solute clearance, contributing to a better understanding of the function of AQP4 in neurofluid dynamics.

## Introduction

The glymphatic system hypothesis was proposed by Iliff et al.^1^. Since then, neurofluid dynamics have received considerable attention^2^. This concept proposes that cerebrospinal fluid (CSF) flows in and out of the brain’s interstitial space through the perivascular spaces, and the circulation and dynamics of neurofluid, including interstitial fluid (ISF), blood, and CSF, contributes to the waste removal from the brain parenchyma. A key protein in this neurofluid dynamics is the aquaporin-4 (AQP4) water channel. AQP4 is abundantly expressed in the endfeet of astrocytes around small vessels, acts as a water-selective channel^3^, and is essential in the clearance of waste products from the brain through neurofluid circulation, especially the exchange of CSF and ISF^1,4^. AQP4 plays a key role in the pathogenesis of several neurological diseases, most notably neuromyelitis optica spectrum disorder (NMOSD). NMOSD is an autoimmune disease in which anti-AQP4 antibodies activate both the complement cascade and DNA damage pathways, promoting inflammatory cell infiltration and resulting in AQP4 depletion, astrocyte death, demyelination, and neuronal loss^5,6^. Furthermore, AQP4 is associated with Alzheimer’s disease^7,8^, Parkinson’s disease^9^, idiopathic intracranial hypertension^10^, and amyotrophic lateral sclerosis^11^, thereby receiving significant attention from a clinical perspective^4^. However, the exact mechanism underlying AQP4’s action has not been fully detailed yet, prompting various preclinical studies.

The function of AQP4 in neurofluid dynamics has also been investigated. In rodent microscopy experiments, the transition of fluorescent CSF tracer into brain parenchyma was reduced in AQP4 knockout (KO) mice, suggesting that AQP4 deficiency reduces CSF-ISF exchange^1,12^. In dynamic contrast-enhanced magnetic resonance imaging (DCE-MRI) with intrathecal tracer, in vivo MR imaging has shown that AQP4 KO mice or acute pharmacologic inhibition of AQP4 using TGN-020 decreased CSF-ISF exchange^13–15^. Furthermore, pharmacologic facilitation of AQP4 using TGN-073 increases CSF-ISF exchange^16^. These results suggest a significant association between AQP4 expression and CSF-ISF exchange. However, this hypothesis has limitations; for instance, the AQP4 KO model is limited to mice and has not been validated in other species.

In contrast, studies on AQP4 function in the clearance of CSF and solutes, such as brain waste products from the cranium, are limited^17^. Several pathways CSF drainage from the subarachnoid space to the extracranium have been reported, including the transnasal route involving the cribriform plate, through which the olfactory nerves pass^18,19^, the perineural route in the perineurium of other cranial nerves (e.g., the optic nerve)^20,21^, and dural lymphatic vessels^22,23^. It is possible that CSF solutes such as brain waste products are also cleared along with CSF through these pathways. Although the association between these pathways and AQP4 remains unexplored, AQP4 is expressed in the olfactory neuroepithelium of rodents^24^, which is an important CSF drainage pathway, suggesting that AQP4 functions in this pathway. AQP4 KO mice exhibited restricted solute translocation along the optic nerve^25^, and AQP4 may also play a role in this perineural route. Therefore, AQP4 expression may also affect the CSF drainage pathway and solute clearance; however, this has not been thoroughly investigated yet.

DCE-MRI with intrathecally administered gadolinium-based contrast agents as a CSF tracer (Gd-tracer) is a valuable method for imaging CSF and solute dynamics, and has been used in rodent experiments to assess CSF-ISF exchange^26^. Compared to fluorescent tracer studies using two-photon microscopy or tissue sections, DCE-MRI is less invasive and comprehensively visualizes intracranial spaces^26^. Furthermore, an evaluation of extracranial structures with an expanded field-of-view (FOV) may be useful for evaluating CSF drainage pathways and solute clearance^27^.

This study aimed to investigate whether AQP4 expression affects CSF solute clearance from the subarachnoid to extracranial spaces, CSF drainage pathways, and intracranial CSF-ISF exchange, using DCE-MRI with intrathecal Gd-tracer in a newly established AQP4 KO rat model.

## Materials and methods

All animal experiments were reviewed and approved by the Animal Research Committee of Osaka University (24-006-042), the Keio University Animal Ethics Committee (A2022-096), and the Institutional Animal Care and Use Committee of Hokkaido University (20–0078), and were performed in accordance with the Japanese Act on Welfare and Management of Animals. The study was reported in accordance with the ARRIVE guidelines (https://arriveguidelines.org).

### Establishment of AQP4 KO rat models

AQP4 knockout rats (LEW-*Aqp4*^*em1Mysi*^) were established during the development of LEW-*AQP4*^*em2Mysi*^ via genome editing using the CRISPR-CAS9 method^28^. In these rats, 295-bp nucleotides spanning the last 292 amino acids of exon 1 and three nucleotides of the flanking intron were deleted (Supplemental Figure S1A). Conventional PCR performed using the primer set 5ʹ-TCCTCAATGTCCCTTCCACTGGAGGCTTCTCATGC-3ʹ and 5ʹ-ACCCAGGACCCCATGCGTGCAGGCAAGCGGTCTGC-3ʹ showed an amplification of wild-type (634 bp) and knockout (339 bp) alleles (Supplemental Figure S1B).

### Western blotting

The cerebella of wild-type (AQP4+/+, WT, *n* = 2) and AQP4 KO (AQP-/-, KO, *n* = 2) rats were homogenized in 250 mM sucrose on ice and centrifuged at 500 × g at 4˚C for 10 min. The supernatant was centrifuged at 12,000 × g at 4˚C for 15 min. Subsequently, the precipitates were solubilized in a buffer containing 20 mM Tris-HCl (pH 7.5), 1 mM EDTA, 1% Triton X-100, and complete protease inhibitor cocktail tablets (Roche Diagnostics, Indianapolis, IN, USA). The concentration of the lysates was measured using a bicinchoninic acid protein assay kit (Pierce, Rockford, IL, USA). Next, 20 mg of protein was subjected to SDS-PAGE on a 12.5% gel containing 3 mol/L urea; the proteins were then transferred to a polyvinylidene difluoride membrane (Millipore, MA, USA) and blocked with 5% skim milk (Becton-Dickinson, Franklin Lakes, NJ, USA) in PBS containing 0.05% Tween 20 (FUJIFILM Wako Pure Chemical Corporation). Signals were visualized using the Immobilon Western Chemiluminescent HRP Substrate (Millipore, MA, USA) and detected using an Image Quant LAS system (GE Healthcare, Waukesha, WI, USA). The antibodies used were rabbit anti-AQP4 extracellular antibody (Sigma, St. Louis, MO, USA, 1:1000), goat HRP-labeled anti-rabbit IgG (R&D Systems, Inc., Minneapolis, MN, USA, 1:5000), and HRP-labeled mouse anti-β-actin antibody (Abcam Limited, Cambridge, UK, 1:10,000).

### Immunohistochemistry

One WT rat and one KO rat were each subjected to perfusion fixation with 4% paraformaldehyde and the brain, kidneys, and skeletal muscles were removed under deep anesthesia induced by isoflurane inhalation. Paraffin-embedded tissue section (5 μm thick) were prepared and stained with an anti-AQP4 antibody (H-80; Santa Cruz Biotechnology, Santa Cruz, CA, USA).

### Reverse transcription polymerase chain reaction (RT-PCR)

Total RNA was extracted from the cerebellum of one WT rat and one KO rat using Isogen (Nippon Gene Co., Ltd., Toyama, Japan). First-strand cDNAs were synthesized using SuperScript VILO Master Mix (Thermo Fisher Scientific, Waltham, MA, USA), according to the manufacturer’s instructions. Conventional PCR was performed using GoTaq Master Mix (Promega, Madison, WI, USA) at 94 °C for 1 min, 65 °C for 1 min, and 72 °C for 3 min (35 cycles).

### Animal preparation for DCE-MRI

Six male AQP4 KO rats (9–10 weeks old, 253–297 g) and eight male WT rats (Lew/Crlcrlj) (9–10 weeks old, 294–312 g; Jackson Laboratory Japan, Kanagawa, Japan) were used. All rats were group housed (up to three animals/cage) under standardized conditions (22–24 °C, light/dark cycle: 12 h/12 h) with *ad libitum* access to food and water.

For pretreatment, anesthesia was induced with 4% isoflurane (Pfizer Japan, Tokyo, Japan) and maintained with 2% isoflurane mixed with ambient air using a small-animal anesthesia machine (SN-487; SHINANO Manufacturing, Tokyo, Japan). Additionally, 2 mg/kg of butorphanol (Vetorphale; Meiji Seika Pharma, Tokyo, Japan) was injected subcutaneously for analgesia. Cisterna magna (CM) cannulation was performed as described previously^29^. The rats were transferred to a stereotaxic instrument with their heads immobilized using ear and tooth bars in a prone position. The median posterior cervical skin was incised to expose the dura overlying the CM. Following a small durotomy with a 23-gauge needle, a sharpened-tip glass cannula (GC-1.5, OD 1.5 mm, ID 0.9 mm; Narishige, Tokyo, Japan) with an MRI-compatible single guide cannula (C313; Protech International Inc., San Antonio, TX, USA) was inserted approximately 1 mm into the CM, fixed with cyanoacrylate, and the incision site was sutured.

### DCE-MRI experiment

All rats were prepared before being transferred to an MRI scanner. The glass cannula was connected to a polyethylene tube (KN-392 SP-45, OD 0.96 mm, ID 0.58 mm; Natsume Seisakusho Co. Ltd., Tokyo, Japan) loaded with Gd-tracer just before scanning.

**Fig. 1:**
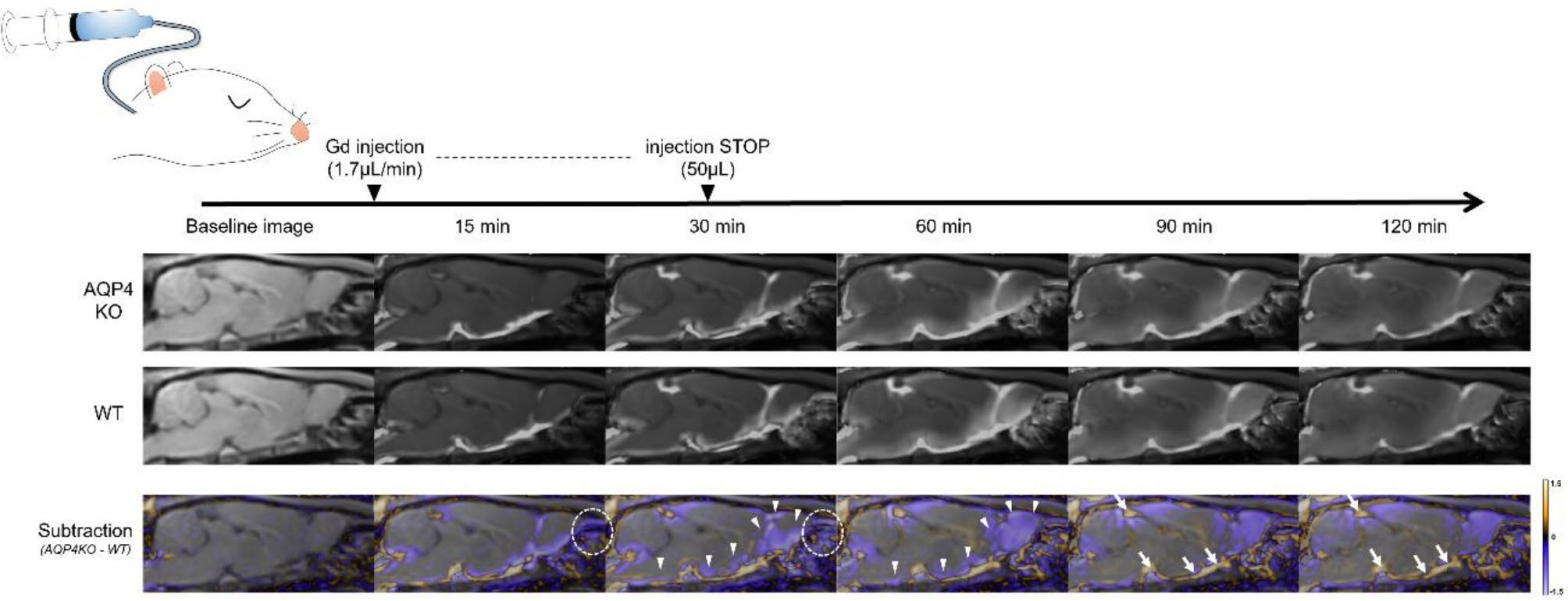
Schematic diagram of the experiment and average and subtraction images of intrathecal Gd-tracer changes over time in each group. The signal ratio maps that overlay post-/pre-contrast signal ratio images on the T1-weighted sagittal images of the WT and KO groups are shown in grayscale in the top and middle rows. Each signal ratio map represents the average data for the five rats in each group. The subtraction images, generated by subtracting the signal ratio maps of the WT group from those of the KO group and converting them into color maps with a range of − 1.5 to 1.5, are shown in the bottom row. In the subtraction images, violet indicates a lower signal (i.e., a lower tracer distribution) in the KO group than that in the WT group, and orange indicates the opposite. The KO group had a lower Gd-tracer distribution in the nasal cavity than the WT group (circles). In contrast, the KO group showed a higher Gd-tracer distribution in the ventral and rostral CSF spaces (arrows) than the WT group. In the KO group, the Gd-tracer distribution in the brain surface parenchyma was lower than that in the WT group (arrowheads). Gd, gadolinium-based contrast agents; KO, aquaporin 4 knockout; WT, wild type.

MRI data were acquired using a 3.0-T clinical scanner (Magnetom Prisma; Siemens Healthcare, Erlangen, Germany) with a 45-mm diameter 8-channel phased array receiving coil (Takashima, Tokyo, Japan). A 3D T2-weighted image, using a 3D sampling perfection with application-optimized contrasts sequence with different flip angle evolutions (SPACE), was acquired for anatomic evaluation (TR/TE = 2500 ms/317 ms; FOV = 76.8 × 65.6 × 70.4 mm^3^; acquisition matrix = 192 × 148 × 176; reconstruction matrix = 192 × 164 × 176; reconstruction resolution = 0.4 × 0.4 × 0.4 mm^3^; number of excitations = 1; scan time per phase = 4 min 44 s). Subsequently, a 3D T1-weighted image, using a 3D volumetric interpolated breath-hold examination (VIBE) sequence, was acquired in the sagittal plane (TR/TE = 8.72 ms/3.29 ms; flip angle = 15°; FOV = 76 × 66.5 × 51.2 mm^3^; acquisition matrix = 192 × 168 × 256; reconstruction matrix = 384 × 336 × 256; reconstruction resolution = 0.2 × 0.2 × 0.2 mm^3^; number of excitations = 2; scan time per phase = 4 min 54 s; number of repetitions = 28; total scan time = 137 min 12 s). After capturing three phases of pre-contrast T1-weighted images, 50 mmol/L of Gd-HP-DO3A (ProHance; Eisai, Tokyo, Japan) diluted with normal saline was intrathecally infused at a rate of 1.7 µL/min, with a total amount of 50 µL, using an MRI-compatible microinjector (PHD2000 infusion; Harvard Apparatus, Holliston, MA, USA). Twenty-five phase images were acquired after Gd-tracer administration started. A schematic of the experiment is shown in Fig. 1. Two rats with Gd-tracer leakage and two rats with failed data acquisition due to mechanical error were excluded, and five rats in each group were included in the analysis.

Respiration and body temperature were continuously monitored during MRI measurements using an MRI-compatible sensor system (Model 1040; SA Instruments, Stony Brook, NY, USA). A custom-made silicone heating system was used to maintain the rectal temperature at 36.5 ± 1.0 °C and respiratory rate at 50–80 breaths/min. At the end of the experiment, the animals were euthanized through an isoflurane overdose.

### Image data processing

Image processing was performed using MATLAB (MATLAB 2022b; The MathWorks, MA, USA), ImageJ (https://imagej.nih.gov/ij/), the SPM12 software (https://www.fil.ion.ucl.ac.uk/spm/), and ANTs (https://stnava.github.io/ANTs)^30^. GPT-4 (https://chatgpt.com/), a large language model, was used to generate and refine the MATLAB codes, and each code was carefully confirmed to function accurately. DCE 3D T1-weighted images were rigidly aligned to the average image of the pre-contrast three phases, and noise reduction was performed in SPM12. The anatomical standard brain was constructed from the 3D T2-weighted images of all rats, and each DCE 3D T1-weighted image was transformed to fit this standard brain in ANTs. Signal intensity changes over time were analyzed, signal ratios relative to the average of the pre-contrast phases were calculated, and the signal ratio map, which overlaid the signal ratios on the T1-weighted anatomical images, was generated for each rat. For a visual comparative assessment between the KO and WT groups, subtraction images were generated by subtracting the average signal ratio map of the WT group from that of the KO group and converted into color maps with a range of − 1.5 to 1.5. In the subtraction images, a positive value (shown in orange) indicated a higher signal (i.e., a higher tracer distribution) in the KO group, whereas a negative value (shown in violet) indicated a lower signal (i.e., a lower tracer distribution) in the KO group than that in the WT group.

**Fig. 2:**
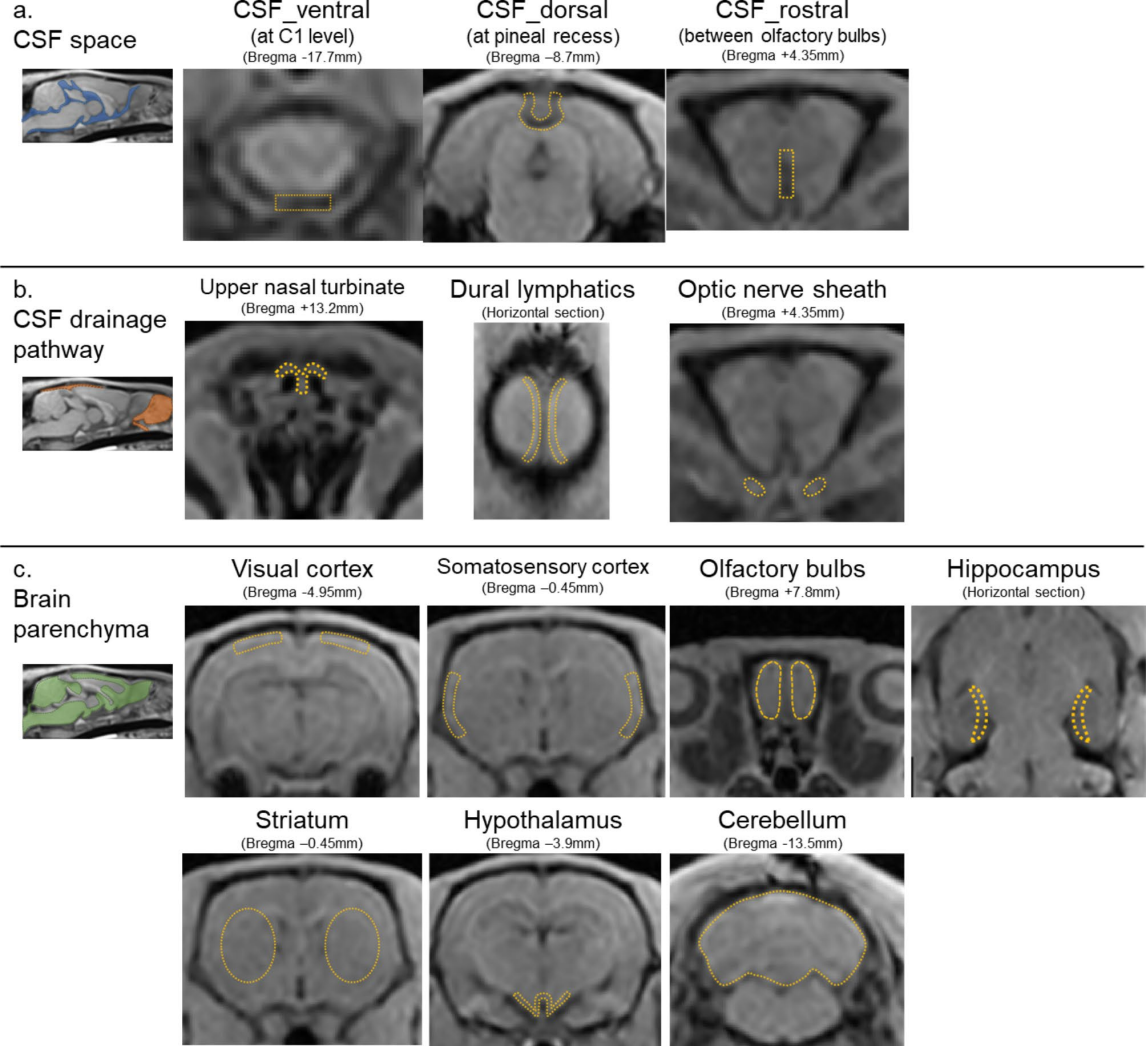
ROI placements depicted by orange dashed lines on T1-weighted images. ROIs were set at ventral, dorsal, and rostral CSF spaces (**a**), the upper nasal turbinate, dural lymphatics, and optic nerve sheaths for the assessment of CSF drainage pathways (**b**), visual cortex, somatosensory cortex, olfactory bulbs, hippocampus, striatum, hypothalamus, and cerebellum for brain parenchyma (**c**). CSF, cerebrospinal fluid; ISF, interstitial fluid; ROI, region of interest.

Regions of interest (ROI) were designed manually on the standard brain and automatically applied and adjusted to each rat’s brain. One researcher defined the ROI, and another researcher verified its validity. ROIs were placed at the following 13 locations (Fig. 2). To assess the Gd-tracer dynamics in the CSF spaces, ROIs were placed in CSF spaces at the C1 level (CSF_ventral), pineal recess (CSF_dorsal), and between the olfactory bulbs (CSF_rostral), based on a previous study of intrathecal tracer dynamics in the CSF spaces^26^. To evaluate the transition of Gd-tracer from the CSF to the extracranial space, ROIs were placed at known CSF drainage pathways: the upper nasal turbinate (UNT), dural lymphatics (DL), and optic nerve sheath (OPT), based on previous reports^31–33^. Furthermore, to evaluate signal changes in the brain parenchyma, indicating Gd-tracer transition from CSF to brain tissue, ROIs were placed at the cerebral cortex (visual area (VIS), somatosensory area (SS)), olfactory bulbs (OLF), hippocampus (HP), striatum (ST), hypothalamus (HT), and cerebellum (CB), referencing related studies and AQP4 expression sites^13,14^.

For each ROI, the signal ratio data were smoothed using the moving average in the MATLAB software. The time-intensity curve (TIC) of the KO and WT groups was plotted with the mean ± standard deviation (SD). In addition, the area under the curve (AUC), arrival time (AT), time-to-peak (TTP), peak signal ratio (PS), and maximum slope (MS) were calculated for the six ROIs to evaluate the CSF spaces and drainage pathways using a modified method of previous research^34^. PS and AUC were evaluated to assess the maximum value and cumulative amount of Gd-tracer distribution, respectively. AT, TTP, and MS were used to assess the time and speed of the Gd-tracer transfer and circulation. AT was defined as the time from the start of intrathecal Gd-tracer injection till the signal intensity increased by more than 10% above the average of the pre-contrast phases. TTP was defined as the interval from the onset of intrathecal Gd-tracer injection till the signal intensity reached its peak. MS was determined as the slope from the arrival of the Gd-tracer to the peak signal intensity.

### Statistical analysis

All statistical analyses were performed using GraphPad Prism 9 (GraphPad Software, San Diego, CA, USA) and the MATLAB software. No statistical approach was used to determine the sample size, consistent with previous reports^13,14^. A statistical comparison of TIC between the KO and WT groups was performed using a two-way analysis of variance (ANOVA). TIC parameters, such as AUC, AT, TTP, PS, and MS, were compared between the two groups using the Mann–Whitney U test, and the obtained p-values were adjusted using Holm correction for each parameter.

All tests were considered statistically significant at *p* < 0.05 (after post hoc correction, if applicable). This was an exploratory study, and not all analyses were corrected for multiple comparisons. All data are presented as means ± SD, unless otherwise noted.

## Results

### Establishment of AQP4 KO rats

To generate AQP4 KO rats, we used the CRISPR-CAS9 technology to obtain rats with a 295-nucleotide deletion at the AQP4 gene locus (Supplemental Fig. S1A). The obtained rats were healthy and bred normally. The rats did not express AQP4 isoforms with the expected molecular mass, as determined via western blotting (Supplemental Figures S1C and S6). Immunohistochemical analysis also demonstrated that the specific immunoreactivity for AQP4 was eliminated in the brain, kidney, and skeletal muscle of the rats (Supplemental Figure S2). The edited allele lacked a 5’-splice site at the boundary of exon 1 and the flanking 3’-intron; therefore, abnormal splicing was expected to occur and produce unusual proteins. We examined the structures of the transcripts derived from the edited alleles (Supplemental Figures S3 and S4). Although some small truncated AQP4 polypeptides were expressed, we confirmed that no functional AQP4 isoforms were expressed in the rats (Supplemental Figure S5).

### DCE-MRI experiment

The dynamics of the intrathecally injected Gd-tracer in the KO (*n* = 5) and WT (*n* = 5) groups are depicted as average images for each group in the sagittal sections of the whole brain displayed in Fig. 1. The Gd-tracer injected into the CM progressively spread through the CSF space from the caudal to the rostral direction, gradually transitioning to the parenchyma of the brain surface and washing out into the extracranial spaces (e.g., the nasal cavity; Fig. 1).

**Fig. 3:**
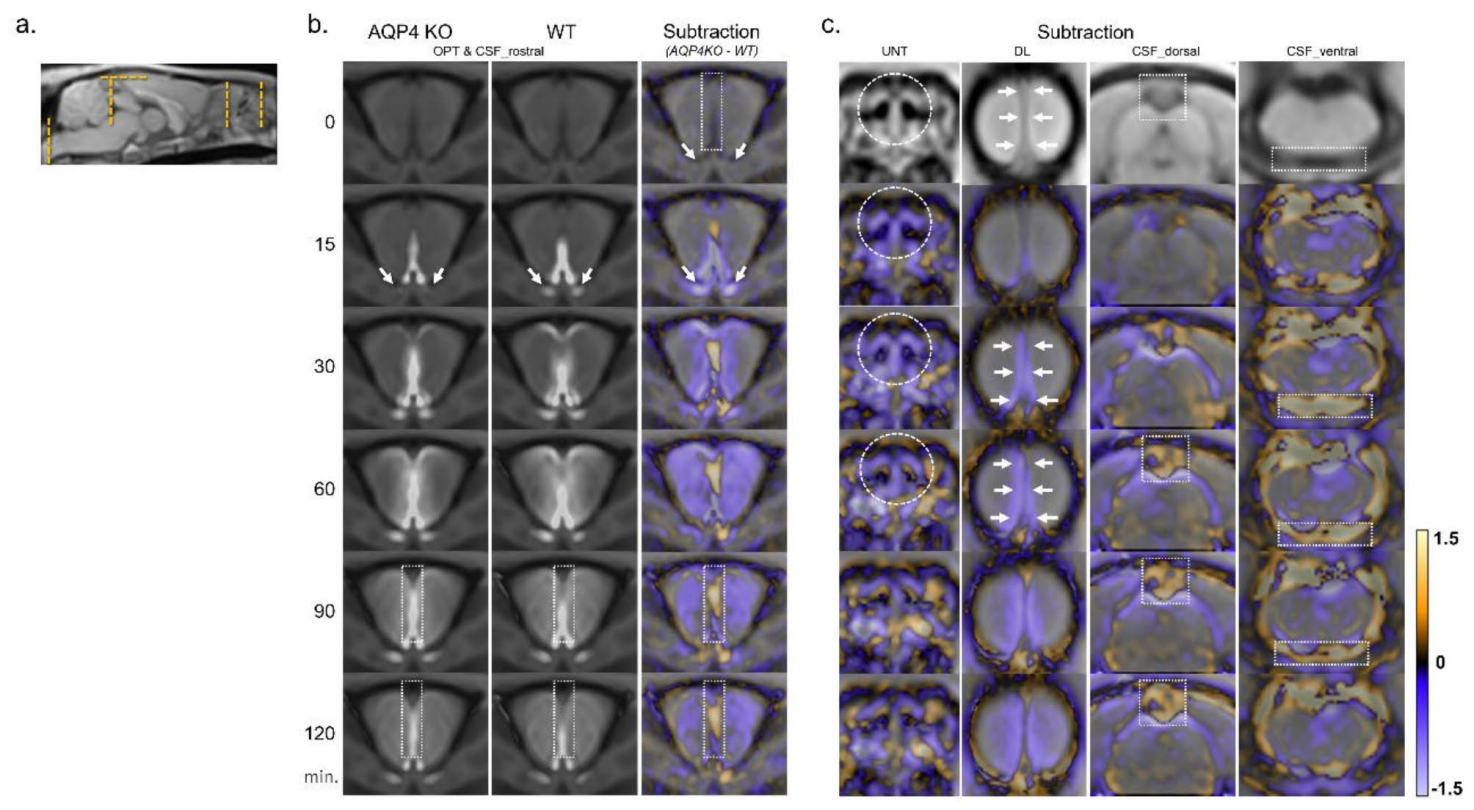
Distribution of the Gd-tracer over time in each representative region for CSF space and drainage pathways. In the forebrain section (**b**), signal ratio maps of the KO and WT groups and subtraction images (generated by subtracting the signal ratio maps of the WT group from those of the KO group and converting them into color maps) are shown. Each signal ratio map represents the average data for the five rats in each group. In the subtraction images, violet indicates a lower tracer distribution in the KO group than that in the WT group, whereas orange indicates the opposite. Each subtraction image is shown in sections of the nasal cavity, dural lymphatics, and dorsal and ventral CSF spaces (**c**). Cross-sectional references are indicated by orange dashed lines (**a**). In the KO group, Gd-tracer distribution along the optic nerve sheath (arrows in B), upper nasal turbinate (dashed circles in c), and dural lymphatics (arrows in c) was reduced compared to that in the WT group. In contrast, the Gd-tracer distribution in the CSF space of the KO group was higher than that of the WT group (dotted rectangles in b and c, respectively). CSF, cerebrospinal fluid; DL, dural lymphatics; Gd, gadolinium-based contrast agents; KO, aquaporin 4 knockout; OPT, optic nerve sheath; UNT, upper nasal turbinate; WT, wild type.

**Fig. 4:**
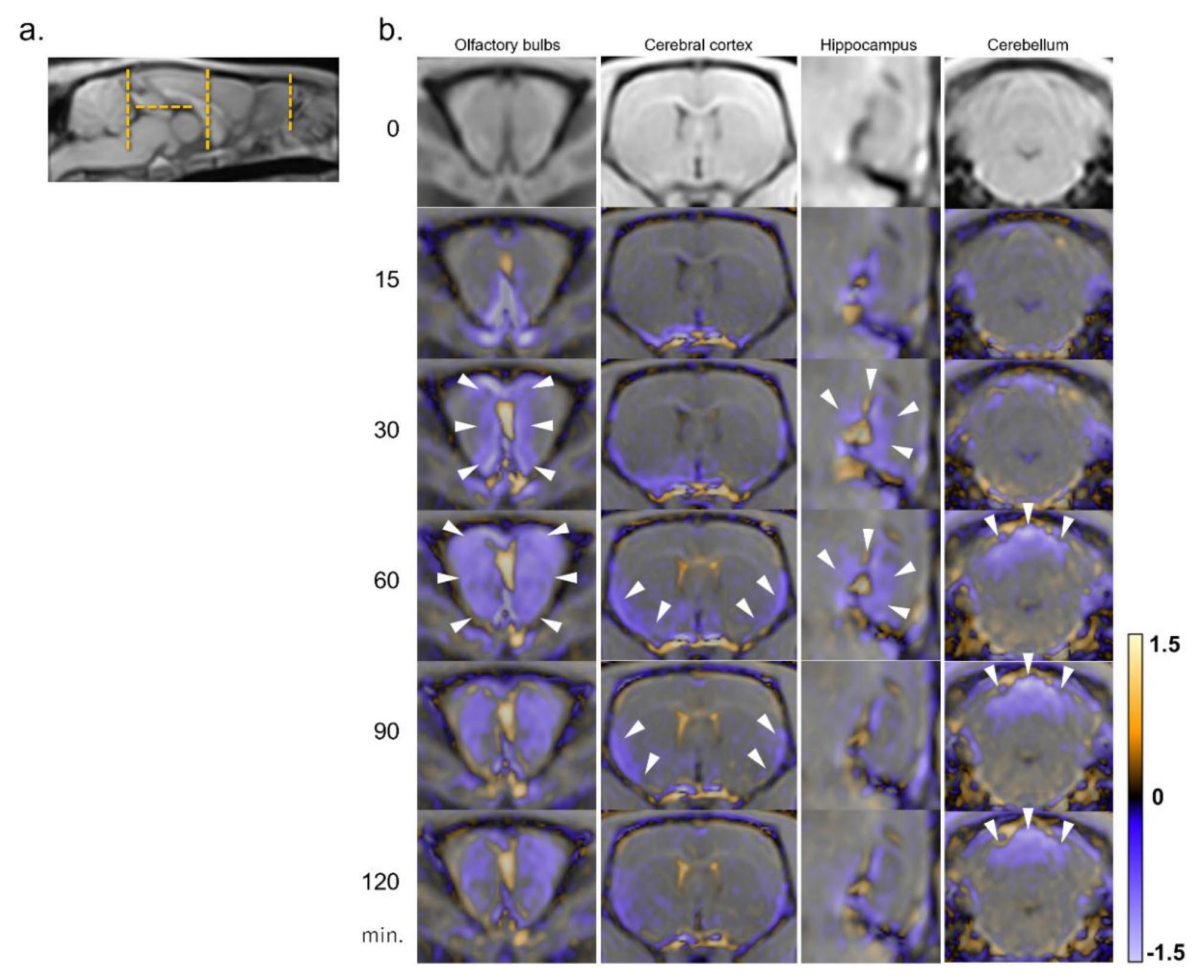
Distribution of the Gd-tracer over time in each representative region for brain parenchyma. (**b**) In sections of the olfactory bulb, cerebral cortex, hippocampus, and cerebellum, subtraction images (generated by subtracting the signal ratio maps of the WT group from those of the KO group and converting them into color maps) are shown. Cross-sectional references are indicated by orange dashed lines (**a**). The KO group showed lower Gd-tracer distribution in the olfactory bulb, ventral and lateral cerebral cortices, hippocampus, and dorsal cerebellum than the WT group (arrowheads). CSF, cerebrospinal fluid; Gd, gadolinium-based contrast agents; KO, aquaporin 4 knockout; WT, wild type.

The focused cross-sectional images captured over time for each ROI are provided in Figs. 3 and 4. The signal ratio maps of the KO and WT groups and subtraction images at the level of the olfactory bulbs captured over time are shown in Fig. 3b. The subtracted images of other ROIs captured over time are presented in Fig. 3c. In the KO group, Gd-tracer distribution in the CSF drainage pathways—the upper nasal turbinate, dural lymphatics, and optic nerve sheath—was reduced compared with that in the WT group. In contrast, the Gd-tracer distribution in the CSF spaces (ventral, dorsal, and rostral) of the KO group was higher than that of the WT group (Fig. 3b and c). Furthermore, the KO group exhibited a lower Gd-tracer distribution in the brain surface parenchyma, especially in the olfactory bulbs, ventral and lateral cerebral cortices, hippocampus, and cerebellum, than the WT group (Figs. 1 and 4).

**Fig. 5:**
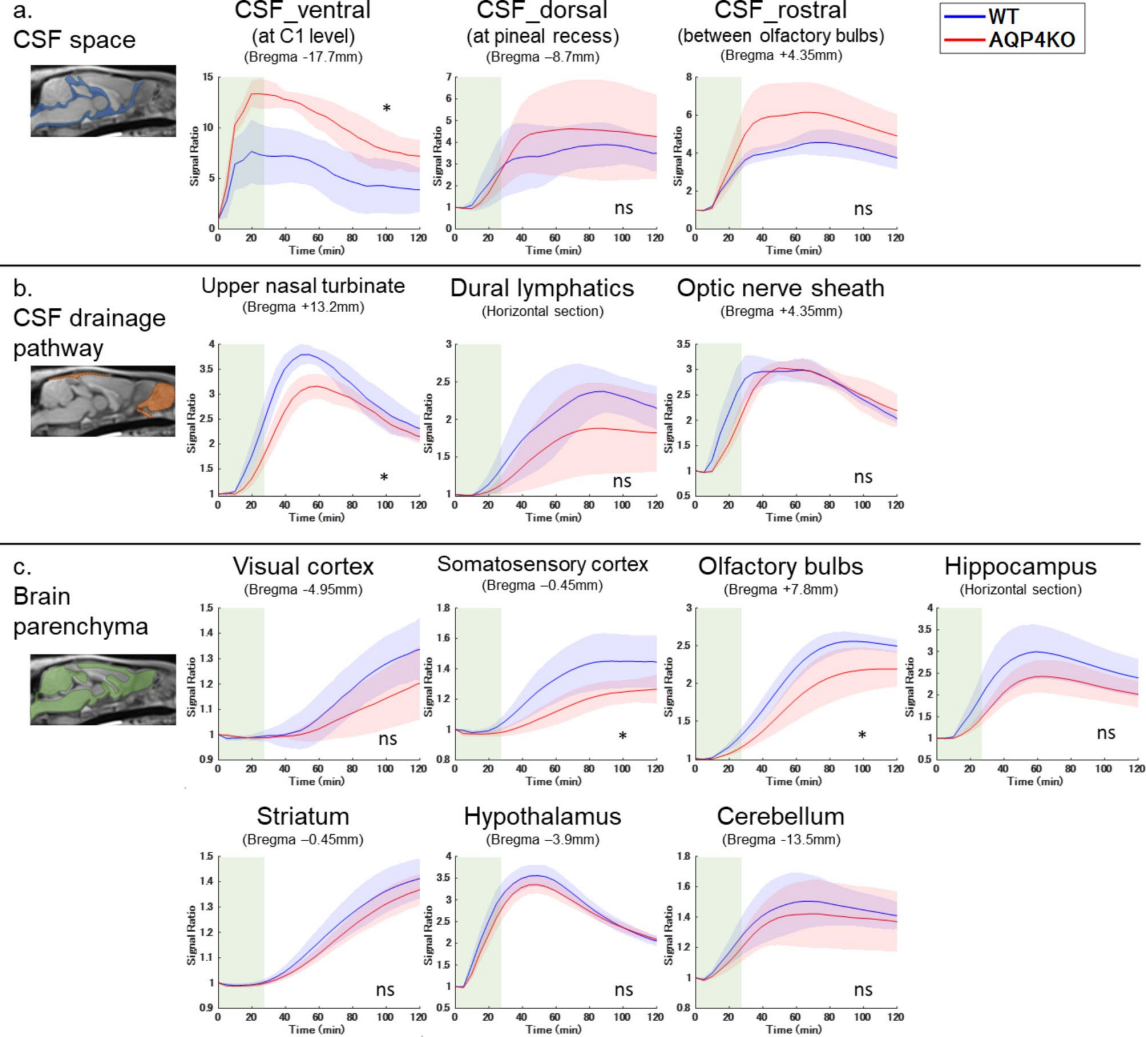
TICs of the signal ratio for each ROI in the WT (*n* = 5) and KO (*n* = 5) groups for (**a**) CSF spaces, (**b**) CSF drainage pathways, and (**c**) brain parenchyma are presented as mean ± SD. The green area represents approximately 30-minute Gd-tracer injection time. The KO group shows higher signal ratios in the CSF space, particularly on the ventral and rostral sides (**a**). In contrast, the signal ratios in the CSF drainage pathway and brain parenchyma were lower in the KO group than in the WT group, especially in the upper nasal turbinate, somatosensory cortex, and olfactory bulbs (**b** and **c**). *, *p* < 0.05, by nonparametric Two-way ANOVA; CSF, cerebrospinal fluid; KO, aquaporin 4 knockout; ns, not significant; ROI, region of interest; TIC, time intensity curve; WT, wild type.

**Fig. 6:**
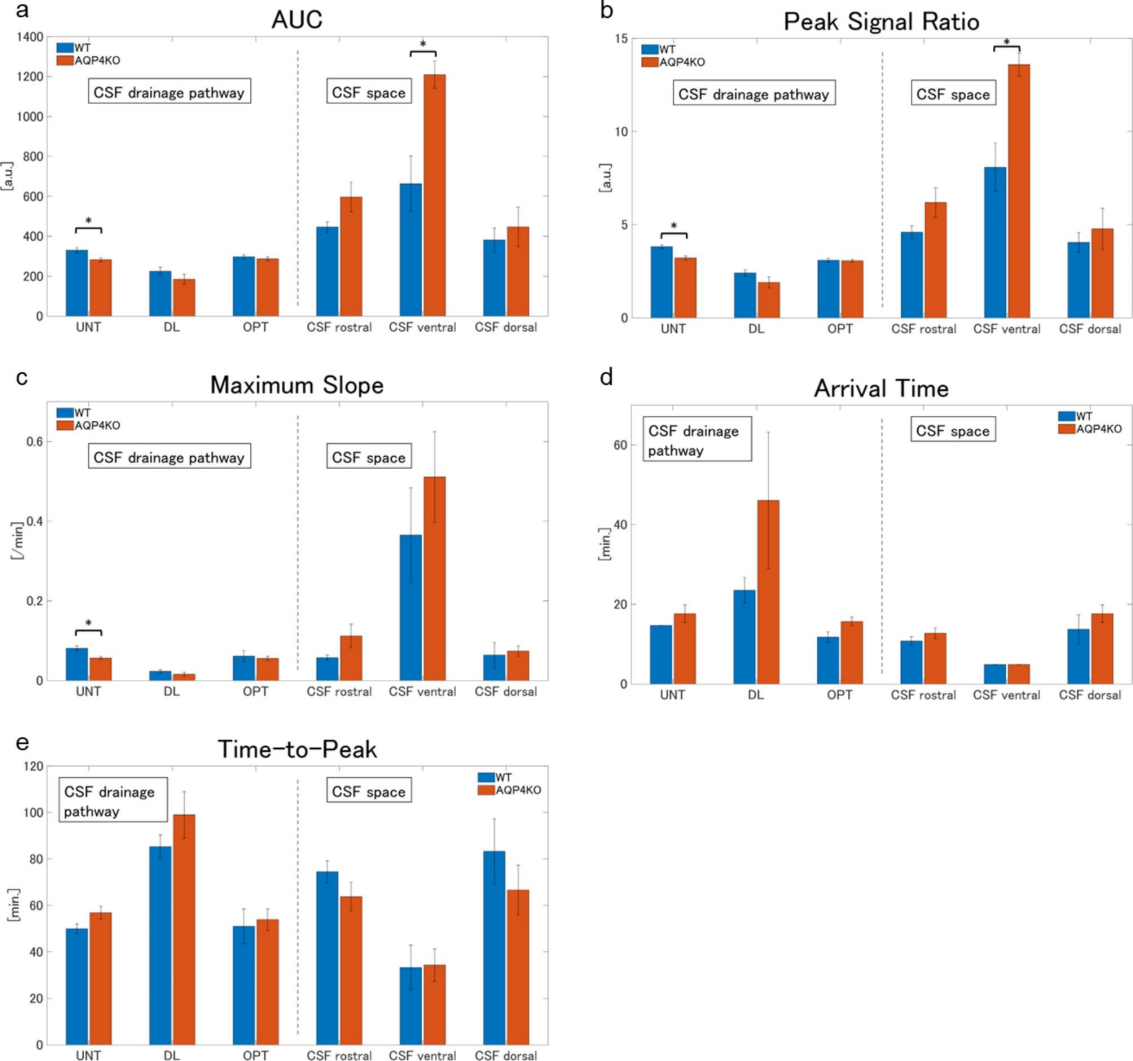
Bar plots of TIC kinetic analysis for CSF drainage pathways (left side) and CSF spaces (right side) in the WT (*n* = 5) and KO (*n* = 5) groups are presented as mean ± SD. The plots are categorized into AUC (**a**), PS (**b**), MS (**c**), AT (**d**), and TTP (**e**). In the KO group, the AUC and MS were higher in each CSF space than those in the WT group; in contrast, they were lower in the CSF drainage pathways, especially the UNT (**a** and **c**). The KO group had a significantly higher PS in the ventral CSF space than the WT group, although the PS was lower in the UNT group (**b**). The AT and TTP of the CSF drainage pathway were longer in the KO group than in the WT group, but the differences were not significant (**d** and **e**). *, *p* < 0.05, by the Mann–Whitney U test after multiple comparison correction; AUC, area under the curve; AT, arrival time; CSF, cerebrospinal fluid; DL, dural lymphatics; KO, aquaporin 4 knockout; MS, maximum slope; OPT, optic nerve; PS, peak signal ratio; UNT, upper nasal turbinate; TTP, time to peak; WT, wild type.

The TIC analysis for each ROI is presented in Fig. 5, and the TIC parameter results for the ROIs of the CSF spaces and drainage pathways are presented in Fig. 6. In the CSF space (ventral, dorsal, and rostral), with UNT, DL, and OPT regarded as CSF drainage pathways, the signal ratio gradually peaked and subsequently washed out. The KO group showed higher signal ratios in the CSF space than that in the WT group, especially in the ventral CSF spaces, with two-way ANOVA revealing a significant difference (*p* < 0.05) (Fig. 5a). In the TIC parameter analysis, the AUC was significantly larger in the KO group than in the WT group in the ventral CSF space (WT: 662.9 ± 277.0, KO: 1209.2 ± 135.7, adjusted *p* = 0.048), and similar differences were observed in the rostral (WT: 446.2 ± 52.09, KO: 596.5 ± 148.5, adjusted *p* = 0.22) and dorsal (WT: 381.3 ± 120.4, KO: 447.2 ± 195.9, adjusted *p* = 0.45) CSF space, although not significant (Fig. 6a). The KO group had a significantly higher PS than the WT group in the ventral CSF space (WT: 8.1 ± 2.6, KO: 13.6 ± 1.2, adjusted *p* = 0.048), whereas a similar trend that was not significant was observed in the rostral (WT: 4.6 ± 0.7, KO: 6.2 ± 1.6, adjusted *p* = 0.16) and dorsal (WT: 4.0 ± 1.1, KO: 4.8 ± 2.2, adjusted *p* = 0.19) CSF spaces (Fig. 6b). No significant differences in AT, TTP, or MS were observed in each ROI of the CSF spaces between the groups (Fig. 6c, d, and e). In contrast, the KO group had lower signal ratios in the CSF drainage pathways than that in the WT group, with notable differences in UNT, as indicated by a two-way ANOVA (*p* < 0.05) (Fig. 5b). The AUC analysis showed a significant difference in UNT (WT: 330.2 ± 26.5, KO: 282.6 ± 18.7, adjusted *p* = 0.04) and an insignificant difference in DL (WT: 224.7 ± 39.6, KO: 185.0 ± 48.1, adjusted *p* = 0.30) and OPT (WT: 297.2 ± 18.8, KO: 286.5 ± 19.9, adjusted *p* = 0.44) (Fig. 6a). PS was significantly lower in the KO group than in the WT group at UNT (WT: 3.8 ± 0.2, KO: 3.2 ± 0.2, adjusted *p* = 0.04), and not significantly lower at DL (WT: 2.4 ± 0.3, KO: 1.9 ± 0.6, adjusted *p* = 0.44) (Fig. 6b). MS was significantly smaller in the KO group than in the WT group at UNT (WT: 0.08 ± 0.01, KO: 0.06 ± 0.01, adjusted *p* = 0.048) (Fig. 6c). AT and TTP were more delayed in the KO group than in the WT group at UNT, DL, and OPT; however, these differences were not significant (Fig. 6d and e).

The signal ratios in the cerebral cortex (OLF, SS, and HP), HT, and CB increased gradually after Gd-tracer injection, subsequently reached a peak, and plateaued or washed out slightly. In the visual cortex and ST, the signal ratio did not reach its peak 120 min after injection (Fig. 5c). In every ROI of the cerebral cortex and deep brain parenchyma, the KO group had a lower signal ratio than the WT group, especially in the OLF and SS, as confirmed by a two-way ANOVA (*p* < 0.05) (Fig. 5c).

Throughout the experiment, the general condition of the rats in both groups remained stable, with no significant differences in respiratory rate or body temperature.

## Discussion

**Fig. 7:**
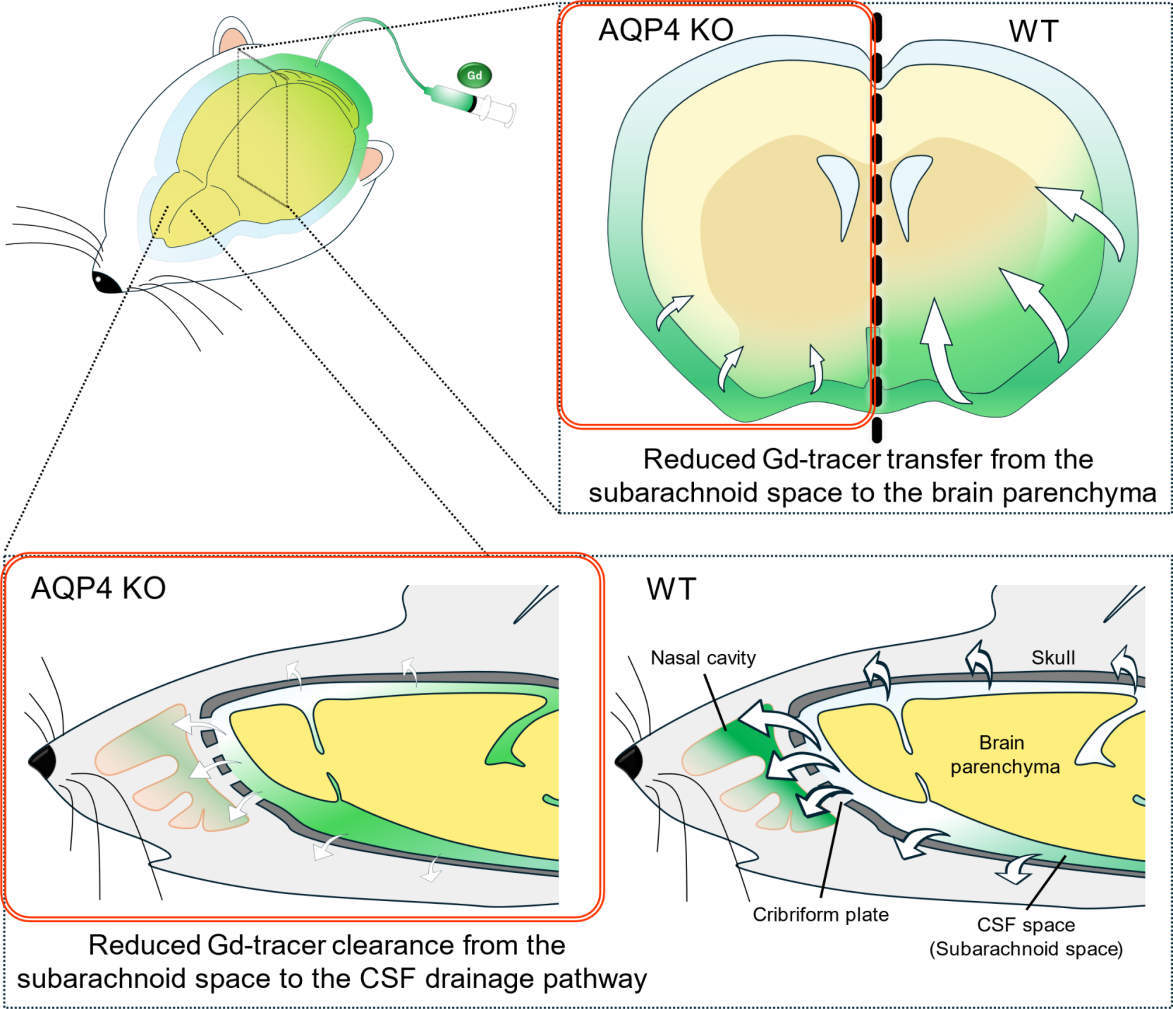
Graphical summary of the main findings of the study. Schematic diagrams illustrating the differences between KO and WT rats in the Gd-tracer transfer to the brain parenchyma and efflux to the extracranial space. CSF, cerebrospinal fluid; Gd, gadolinium-based contrast agents; KO, aquaporin 4 knockout; WT-wild type.

In this study, the intrathecally administered Gd-tracer was transferred to the brain parenchyma and effluxed into extracranial spaces, such as the nasal cavity, as evident via DCE-MRI and consistent with previous reports^26,27,31,33^ (Fig. 7). In addition, AQP4 KO rats showed a higher Gd-tracer distribution in the CSF space compared to WT rats, as seen in signal ratio maps, and with PS and AUC values, indicating a greater maximum and total amount of the Gd-tracer distribution. The difference in tracer distribution was more pronounced on the ventral side. Intrathecal tracers tend to transfer from ventral to rostral in the prone position^26^, making it difficult to detect differences on the dorsal side, where Gd-tracers are less likely to transfer. In contrast, AQP4 KO rats visually showed reduced Gd-tracer transitions to the CSF drainage pathways compared to WT rats, as seen in signal ratio maps, with a significant difference in TIC in the UNT. PS and AUC analysis indicated a lower maximum and total amount of Gd-tracer distribution in the UNT in AQP4 KO rats. Differences in other drainage pathways (DL and OPT) were smaller, aligning with previous reports that the transnasal route is the dominant CSF drainage pathway in rodents^27^, or the UNT may be a suitable site to detect signal changes. AT and TTP analysis showed no significant differences between both groups, however, it might have been undetectable due to the limitations of time resolution of DCE-MRI. These findings, which indicated that AQP4 KO rats exhibited reduced Gd-tracer transition to CSF drainage pathways and increased distribution of Gd-tracer in the CSF space, suggest that the loss of AQP4 may impair solute clearance from the CSF space through CSF drainage pathways (Fig. 7). Previous studies using fluorescent tracers and MR imaging have rarely explored the link between AQP4 functions and the CSF solute clearance and drainage pathways, making this a novel finding. Plá et al. also found that AQP4 KO mice had reduced efflux of fluorescent tracers from the brain parenchyma into the systemic circulation (measured at the femoral vein)^17^, which aligns with our results, despite differences in administration routes and evaluation methods.

The results suggest that AQP4 deficiency may impair CSF solute clearance, though there is no direct evidence linking AQP4 deficiency to specific molecular changes in CSF dynamics, leaving the mechanisms speculative. One hypothesis, based on a study of CSF drainage through the cribriform plate, notes that AQP4 is highly expressed in the olfactory nerve and neuroepithelium^24^, where its role was previously unclear. AQP4 at this site could regulate CSF drainage through the transnasal pathway, and these findings may indirectly support this. AQP4 also facilitates the glymphatic function of the optic nerve^33,35^. For instance, Wang et al. reported that Aβ clearance from the retina and along the optic nerve was reduced in AQP4 KO mice^25^. We also focused on this pathway. However, in this study, the change in Gd-tracer transition along the optic nerve in AQP4 KO rats was not significant, with no corresponding correlation. As an alternative hypothesis, we speculated that the reduction in CSF production and turnover due to AQP4 loss may have resulted in impaired CSF solute clearance, because AQP4 could potentially affect CSF production. For instance, Igarashi et al. reported that water influx to the CSF from the blood was mediated by AQP4^36^, and Trillo-Contreras et al. reported that AQP4 deletion altered CSF drainage and ventricular compliance^37^. However, Gomolka et al. found that AQP4 has no effect on CSF production^13^. The relationship between CSF production and AQP4 remains controversial, and this hypothesis is still speculated.

Our study also showed that the Gd-tracer transition from the CSF space to the brain parenchyma was reduced in AQP4 KO rats. This decrease was more pronounced closer to the surface of the brain. This suggests that AQP4 deletion decreases CSF-ISF exchange, similar to previous reports on AQP4 inhibitors or AQP4 KO mice^12–14^. However, there were some minor discrepancies compared to previous studies. For instance, the differences between AQP4 KO and WT rats in this study were generally less noticeable than those observed in Takano et al.’s study using AQP4 inhibitors^14^. This discrepancy with the study on AQP4 inhibitors suggests that the acute or chronic loss of AQP4 function may have affected the results. Zhang et al. reported that AQP4 KO mice had an increase in capillary density than WT mice and hypothesized that this will compensate for the reduced water exchange due to chronic AQP4 deletion^38^. Hence, compensatory mechanisms may underlie AQP4 function in chronic AQP4 deletion models. Furthermore, whereas the study by Gomolka et al. on AQP4 KO mice showed a reduced CSF-ISF exchange in the deep brain parenchyma due to AQP4 deficiency, this study showed no significant changes in the deep brain parenchyma. This discrepancy with the study of AQP4 KO mice may be because of variations in anesthesia, species, Gd-tracer injection protocols, and MR signal sensitivity. In particular, the isoflurane used for anesthesia in this study exhibits low CSF tracer influx into the brain of rodents^39,40^, which may have affected our results. However, even with minor differences in species and methods, our results, which are similar to those of previous reports, support the hypothesis that AQP4 expression affects CSF-ISF exchange.

The newly established AQP4 KO rats in this study showed no obvious symptoms and no general behavioral or coordination deficits, which is consistent with the observations that mice lacking AQP4 generally exhibited no gross anatomical abnormalities in the whole body, including the brain. Some mild functional impairments (e.g., alterations in retinal response to light and hearing loss) were observed in AQP4 KO mice^41,42^, which we have not checked for our AQP4 KO rats in this study. On the other hand, AQP4 KO mice have shown phenotypes significantly different from those of WT mice in some pathological conditions. For instance, Ishida et al. reported that a tau aggregation mouse model lacking AQP4 showed more severe tau deposition and neurodegeneration in the brain compared to the same model with functional AQP4^7^. Manley et al. reported that AQP4 KO mice showed reduced cellular brain edema in models of acute water intoxication and cerebral infarction^41^. In contrast, Papadopoulos et al. reported that vasogenic brain edema worsened in AQP4 KO mice^43^. Thus, AQP4 may play an important role in dealing with undesirable changes in the brain environment by regulating neurofluid dynamics. The involvement in the CSF solute clearance found in this study may be a part of the mechanism for maintaining the homeostasis of neurofluid.

Previous studies using AQP4 KO animal models have been limited to mice^12,13^. Here, we successfully established AQP4 KO rat models for the first time. Our results in AQP4 KO rats were similar to those of previous reports on AQP4 KO mice, especially regarding the association between AQP4 expression and CSF-ISF exchange. We believe that this further validated the reliability of our findings because the same findings were obtained in different animal species, albeit within the same rodent family. Furthermore, using rats, we successfully acquired images on a 3.0-T clinical MRI scanner with a custom-made receiving coil, thereby preserving the spatial resolution and signal-to-noise ratio. This enabled a comprehensive analysis of solute dynamics, including the extracranial spaces, by expanding the FOV and providing new insights into the CSF drainage pathway and AQP4 expression. Therefore, we consider establishing the AQP4 KO rat model to be significant.

This study had several limitations. First, this study used intrathecal Gd-tracer administration in rats, and this created a non-physiological environment, such as volume loading, which may have affected the results. According to previous reports, the intrathecal infusion rate (1.7 µL/min) and volume (50 µL) used in this study may have a small effect on intracranial pressure (ICP)^44^. While ICP and blood pressure were not measured, no changes in respiration rates, body temperatures, or general conditions were observed. Given that the DCE-MRI results were partly consistent with earlier studies, the findings likely reflect changes under approximate physiological conditions in rats. However, the CSF drainage pathway and solute clearance system may differ between humans and rodents^45^, and the changes observed in this study may not fully represent human physiology. Further studies are needed to investigate the relationship between AQP4 dysfunction, pathology, and CSF solute clearance in humans. Several human studies using intrathecal Gd-tracer have been conducted^46^, and applying these to disease and pathological conditions could provide valuable insights. Less invasive methods, such as using endogenous tracers (the arterial spin labeling method)^47^, may also be beneficial. Second, we estimated the change of CSF solute clearance from the dynamics of the Gd-tracer; however, it is uncertain whether these results reflect the actual changes in physiological proteins or brain waste products, such as tau and amyloid, because there was no direct evidence linking loss of AQP4 to specific molecular changes in CSF solute clearance. Therefore, the relationship between the signal changes observed in this study and actual CSF solute clearance has not been established, and is an indirect proof. Furthermore, it was not possible to assess how the loss of AQP4, which is a water-selective channel, actually caused the restriction in the dynamics of solute tracers that could not pass through AQP4; therefore, the cause is speculative. To better understand how altered water permeability due to AQP4 affects CSF solute clearance, further studies with a water tracer, such as 17O-H2O or D2O, may be useful^48^. Third, this experiment could not assess changes in other potential factors beyond AQP4, which may have influenced the results. For example, any upregulation or downregulation of another AQP subtype (such as AQP1) could affect the findings. Moreover, changes in factors related to CSF production or drainage—such as blood pressure, intracranial pressure, or capillary densities—in AQP4 KO rats could have contributed. Thus, the results cannot be definitively attributed solely to AQP4 deficiency.

In conclusion, DCE-MRI showed that AQP4 KO rats exhibited reduced efflux of intrathecal Gd-tracer into CSF drainage pathways and increased retention of intrathecal Gd-tracer in the CSF space. These results suggest that AQP4 loss may be one of the factors impairing solute clearance from the CSF space through the CSF drainage pathways, either directly or indirectly.

## Electronic supplementary material

Below is the link to the electronic supplementary material.

Supplementary Material 1

## Data Availability

The datasets are presented in the manuscript and supplementary materials, and available from the corresponding author upon reasonable request.
