# Supplementary material for: Loss of aquaporin-4 impairs cerebrospinal fluid solute clearance through cerebrospinal fluid drainage pathways
Source: Sci Rep. 2024 Nov 14;14:27982. doi: 10.1038/s41598-024-79147-y (PMC11564557; doi:10.1038/s41598-024-79147-y)
Supplement: Supplementary file 1 — Supplementary Material 1 [file 41598_2024_79147_MOESM1_ESM.pdf]

Loss of aquaporin-4 impairs cerebrospinal fluid solute clearance through cerebrospinal fluid drainage pathways

Daisuke Kato[1,2], Hiroyuki Kameda[1,3*], Naoya Kinota[1,2], Takaaki Fujii[1,2], Bai Xiawei[2], Zhou Simi[2], Yoshiki Takai[4,5], Simon Chau[6], Yoshiki Miyasaka[7], Tomoji Mashimo[8], Yoichiro Abe[6*], Masato Yasui[6], Kazuyuki Minowa[3], and Kohsuke Kudo[1,2]

1. Department of Diagnostic and Interventional Radiology, Hokkaido University Hospital, Sapporo, Japan
2. Department of Diagnostic Imaging, Graduate School of Medicine, Hokkaido University, Sapporo, Japan
3. Department of Radiology, Faculty of Dental Medicine, Hokkaido University, Sapporo, Japan
4. Department of Neurology, Tohoku University Graduate School of Medicine, Sendai, Japan
5. Department of Pathology, Tohoku University Hospital, Sendai, Japan
6. Department of Pharmacology, Keio University School of Medicine, Tokyo, Japan
7. Laboratory of Reproductive Engineering, Institute of Experimental Animal Sciences, Osaka University Medical School, Suita, Japan
8. Division of Animal Genetics, Laboratory Animal Research Center, Institute of Medical Science, The University of Tokyo, Tokyo, Japan

E-mail: h_kameda@med.hokudai.ac.jp (Hiroyuki Kameda), yoabe@a6.keio.jp (Yoichiro Abe)

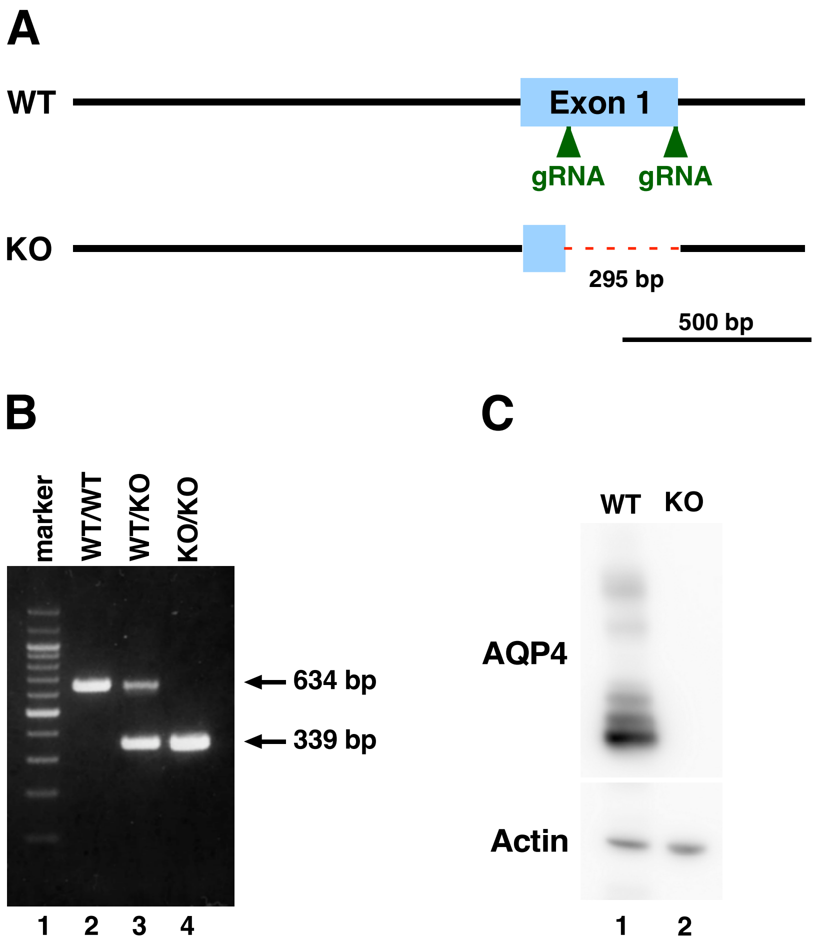

Supplemental Figure S1. Establishment of AQP4 KO rats.
(**A**) A schematic illustration depicting the genome editing strategy and the resulting genomic structure of AQP4 KO rats. An exon is represented by a blue box. A deleted part is represented by a red dotted line. (**B**) A method of genotyping for the AQP4 KO rats using PCR. Single 634-bp and 339-bp bands were amplified from genomic DNA extracting from homozygous wild-type (lane 2) and KO (lane 4) rats, respectively, and both bands were observed in heterozygotes (lane 3). A pattern of 100-bp ladder is also shown (lane 1) (**C**) Expression of AQP4 (upper panel) and actin (lower panel) in the cerebellum of wild-type (lane 1) and AQP4 KO rats (lane 2) determined by Western blotting. The original blots are presented in Supplemental Figure S6.

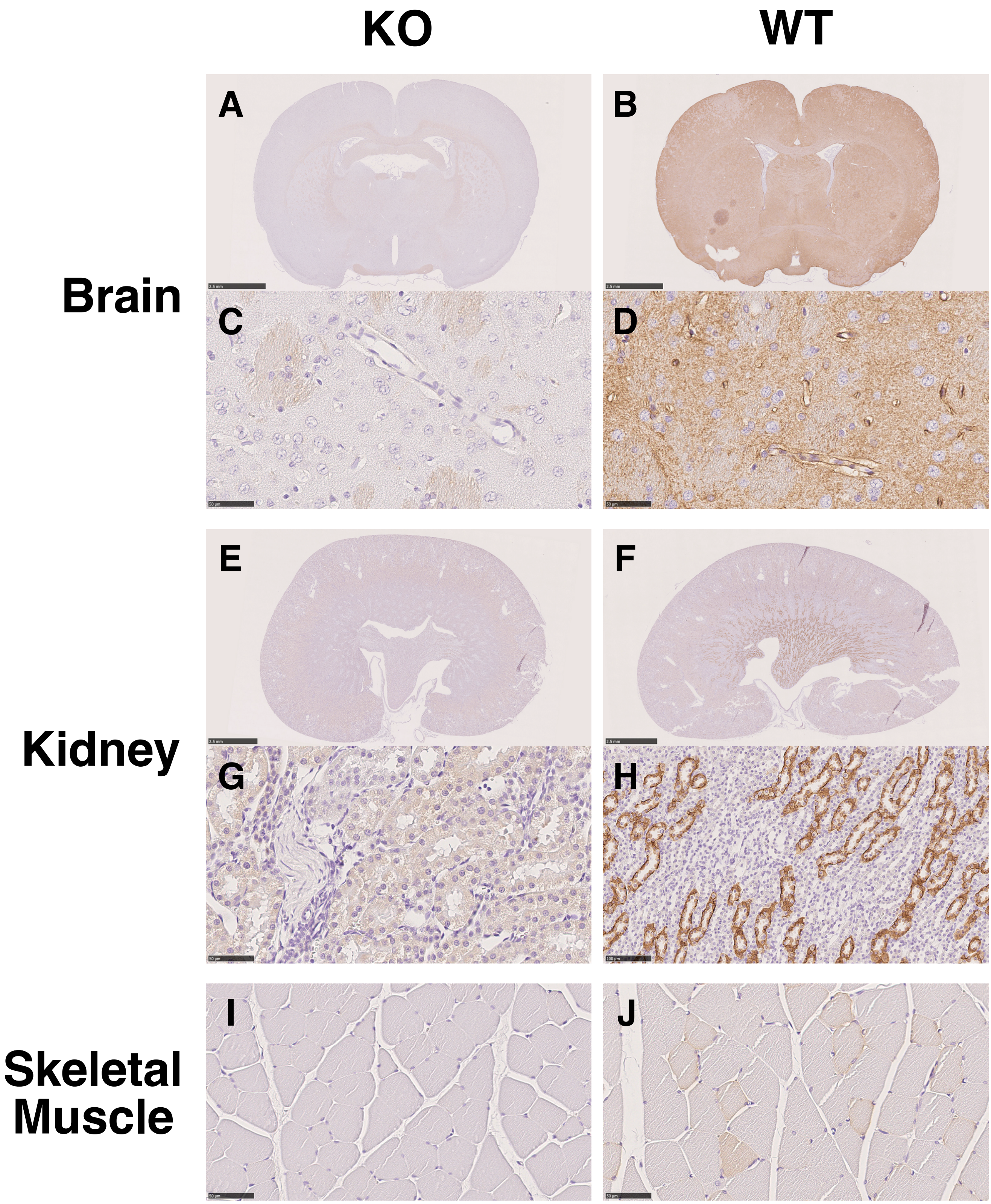

Supplemental Figure S2. Immunohistochemical analysis of AQP4 KO rats.
Paraffin-embedded sections of brains (**A-D**), kidneys (**E-H**) and skeletal muscles (**I, J**) of KO (**A, C, E, G, and I**) and wild-type (**B, D, F, H, and J**) rats were stained by anti-AQP4 antibody. Bars = 2.5 mm (**A, B, E, and F**) or 50 µm (**C, D, G, H, I, and J**)

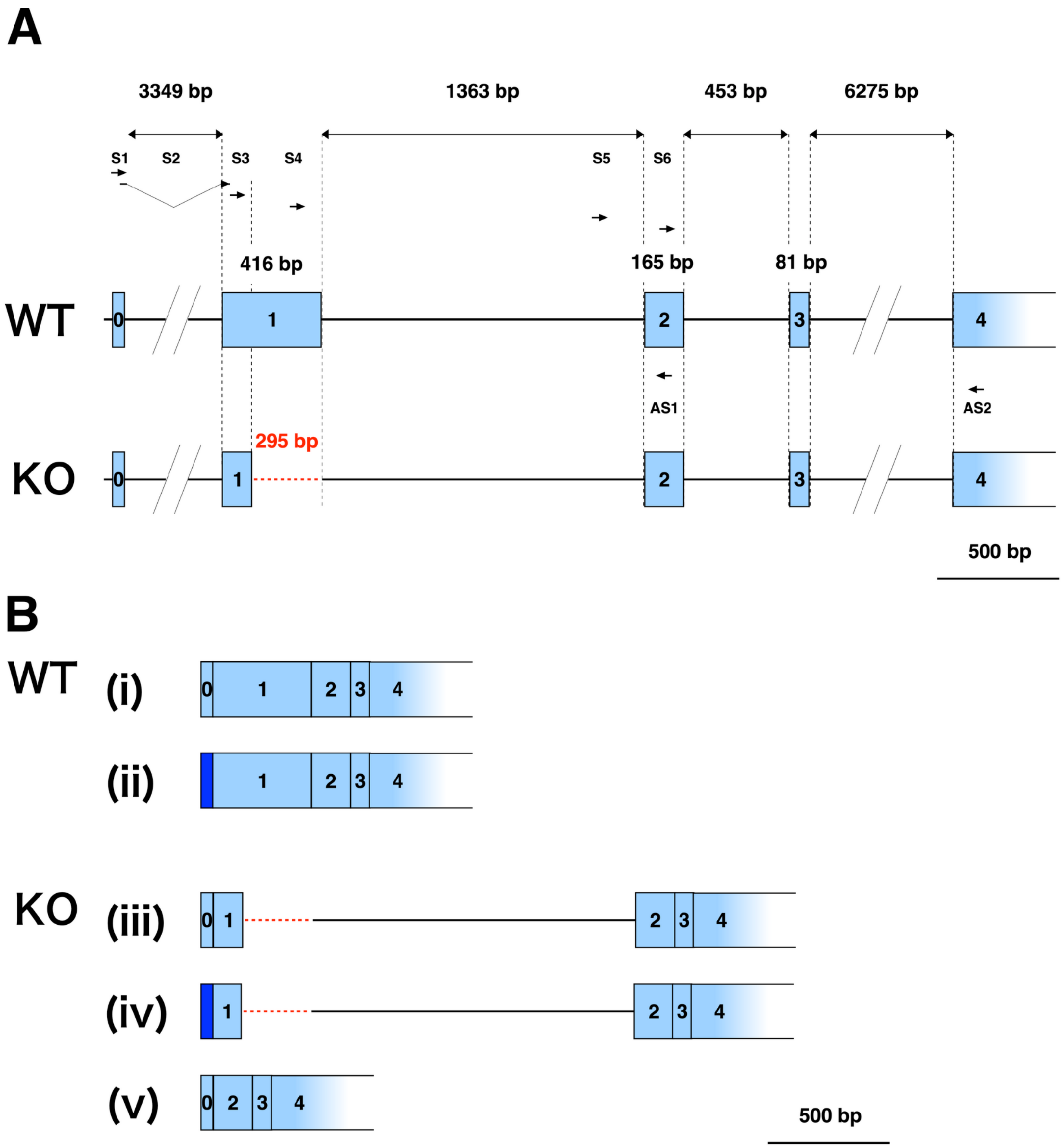

Supplemental Figure S3. Analysis of the structure of transcrtipts expressed in AQP4 KO rats. (**A**) A schematic illustration that compares the structure of the genomic DNA of AQP4 KO rats with that of wild type. Exons are represented by blue boxes. A 295-bp deleted part in the genomic DNA of KO rats is represented by a red dotted line. Primers used for PCR are represented as arrows. Their sequences are shown in Supplemental Table 1. Sizes of possible transcripts are shown in Supplemental Table 2. (**B**) Schematic illustrations of structure of transcripts possibly expressed in KO rats (iii - v) comparing with that of wild type (i and ii). Since the knockout allele lacks a 5'-splice site at the boundary of exon 1 and flanking 3'-intron, it is possible that the remaining part of exon 1, the following intron, and exon 2 as a large exon (iii and iv) or the remaining part of exon 1 and its flanking introns are recognized as an intron (v).

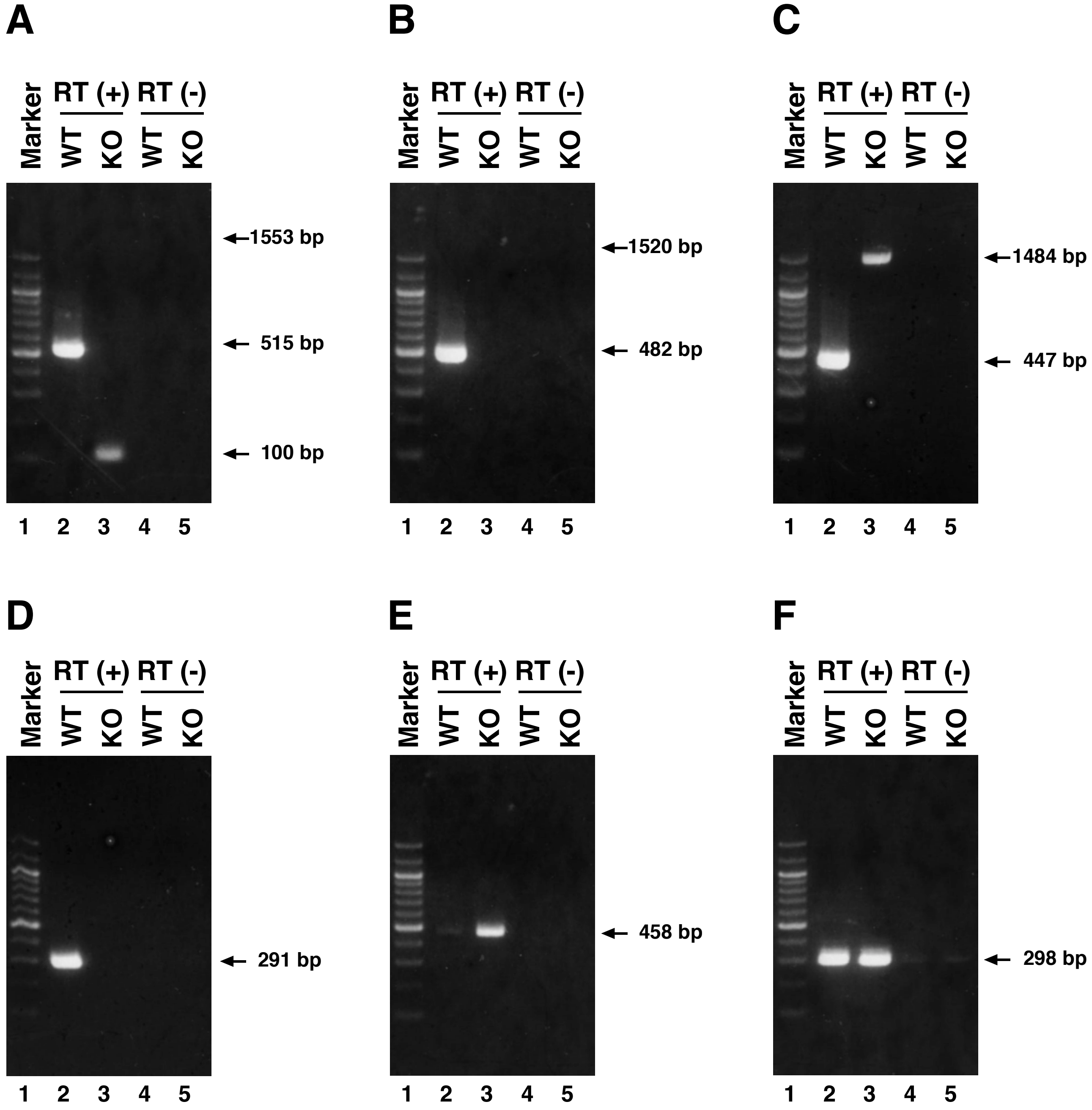

Supplemental Figure S4. Detection of transcripts expressed in the cerebellum of AQP4 KO rats by RT-PCR.

Total RNA extracted from cerebella of wild type (lanes 2 and 3) and knockout (lanes 3 and 5) rats were subjected to reverse transcription reaction in the presence (lanes 1 and 2) or absence (lanes 4 and 5) of reverse transcriptase followed by PCR reaction using primer sets S1-AS1 (**A**), S2-AS2 (**B**), S3-AS1 (**C**), S4-AS1 (**D**), S5-AS2 (**E**) and S6-AS2 (**F**) (Supplemental Figure S3). No amplification was observed when reverse transcriptase was absent in the reaction mixtures (lanes 4 and 5), confirming that no amplification derived from genomic DNA was detected. A pattern of 100-bp ladder is also shown (lane 1).

(**A**) A shorter (100 bp) fragment was amplified in KO by primer set S1-AS1. Cloning and sequence of this fragment revealed that in KO rats, there exists a transcript in which exon 0 is directly spliced to exon 2. On the other hand, this primer set did not amplify a 1553-bp band, indicating lack of transcript iii shown in Supplemental Figure S3B. (**B**) No band was detected in KO rats by primer S2, which recognizes the junction between exons 0 and 1, indicating that there is no transcript in which exon 0 is conected to exon 1, confirming the lack of transcript iii. (**C**) A larger band (1484 bp) was amplified in KO by primer set S3-AS1, indicating that there is an insertion between the remaining portion of exon 1 and exon 2. (**D**) No band was detected in KO rats by primer S4, which recognizes a sequence deleted in KO rats. (**E**) A 458-bp band was amplified in KO rats by primer set S5-AS2, confirming that a transcript in KO rats includes an intron flanked by exon 2. (**F**) A significant level of the band amplified by primer set S6-AS2, which amplifies between exons 2 and 4, was observed in KO rats. Taken together, it is highly likely that transcripts iv and v are dominantly expressed in AQP4 KO rats.

A

```
WT  23 Met Val Ala Phe Lys Gly Val Trp Thr Gln Ala Phe Trp Lys Ala Val Thr Ala Glu Phe 42
KO   1 Met Val Ala Phe Lys Gly Val Trp Thr Gln Ala Phe Trp Lys Ala Val Thr Ala Glu Phe 20

       Transmembrane domain 1
WT  43 Leu Ala Met Leu Ile Phe Val Leu Leu Ser Val Gly Ser Thr Ile Asn Trp Gly Gly Ser 62
KO  21 Leu Ala Met Leu Ile Phe Val Leu Leu Arg Glu Leu Phe Cys Ser Tyr Phe Cys Ala Arg 40

                                        Transmembrane domain 2
WT  63 Glu Asn Pro Leu Pro Val Asp Met Val Leu Ile Ser Leu Cys Phe Gly Leu Ser Ile Ala 82
KO  41 Thr Leu Arg Ala His Lys Leu Cys Arg Lys Ile Gly Gly Leu Arg Gly Thr His Asn Thr 60

WT  83 Thr Met Val Gln Cys ...
KO  61 Thr Gly Leu Gln Ser 65
```

B

```
WT   1 Met Ser Asp Gly Ala Ala Ala Arg Arg Trp Gly Lys Cys Gly Pro Pro Cys Ser Arg Glu 20
KO   1 Met Ser Asp Gly Ala Ala Ala Arg Arg Trp Gly Phe Met Glu Thr Ser Leu Leu Ala Met 20

WT  21 Ser Ile Met Val Ala ...
KO  21 Gly Ser Trp Trp Ser 25
```

C

```
                                                           Transmembrane domain 6
WT 224 Met Gly Asn Trp Glu Asn His Trp Ile Tyr Trp Val Gly Pro Ile Ile Gly Ala Val Leu 243
KO   1 Met Gly Asn Trp Glu Asn His Trp Ile Tyr Trp Val Gly Pro Ile Ile Gly Ala Val Leu  20

WT 244 Ala Gly Ala Leu Tyr Glu Tyr Val Phe Cys Pro Asp Val Glu Leu Lys Arg Arg Leu Lys 263
KO  21 Ala Gly Ala Leu Tyr Glu Tyr Val Phe Cys Pro Asp Val Glu Leu Lys Arg Arg Leu Lys  40

WT 264 Glu Ala Phe Ser Lys Ala Ala Gln Gln Thr Lys Gly Ser Tyr Met Glu Val Glu Asp Asn 283
KO  41 Glu Ala Phe Ser Lys Ala Ala Gln Gln Thr Lys Gly Ser Tyr Met Glu Val Glu Asp Asn  60

WT 284 Arg Ser Gln Val Glu Thr Glu Asp Leu Ile Leu Lys Pro Gly Val Val His Val Ile Asp 303
KO  61 Arg Ser Gln Val Glu Thr Glu Asp Leu Ile Leu Lys Pro Gly Val Val His Val Ile Asp  80

WT 304 Ile Asp Arg Gly Asp Glu Lys Lys Gly Lys Asp Ser Ser Gly Glu Val Leu Ser Ser Val 323
KO  81 Ile Asp Arg Gly Asp Glu Lys Lys Gly Lys Asp Ser Ser Gly Glu Val Leu Ser Ser Val 100
```

Supplemental Figure S5. Polypeptide sequences translated from transcripts in AQP4 KO rats.
(**A**) A polypeptide encoded by transcript iv in Supplemental Figure S3B. (**B**) A polypeptide encoded by transcript v in Supplemental Figure S3B. (**C**) Since the initiation codon for the M1 isoform located in exon 0 is not perfectly match for the Kozak consensus sequence and therefore, the 224th methionine could alternatively function as an initiation codon encoding a 100-bp truncated AQP4 having the sixth transmembrane and the C-terminal domains. Amino acid sequences unrelated to AQP4 caused by truncation of exon 1 or unusual splicing in KO rats are indicated in red. Transmembrane domains of wild-type AQP4 are indicated with lines according to Hiroaki et al., (2006).

A

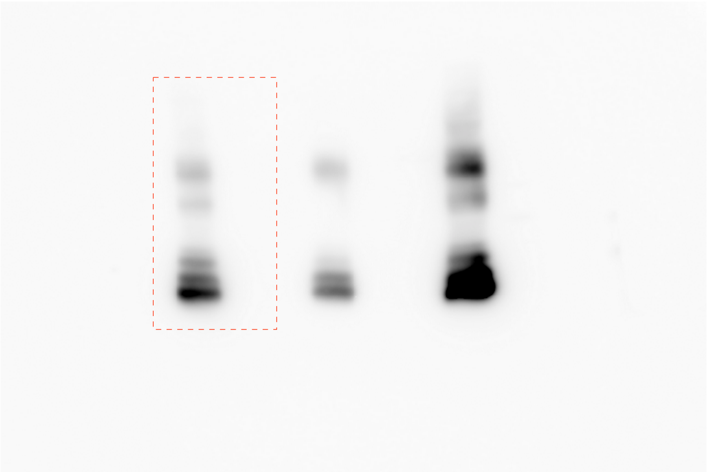

B

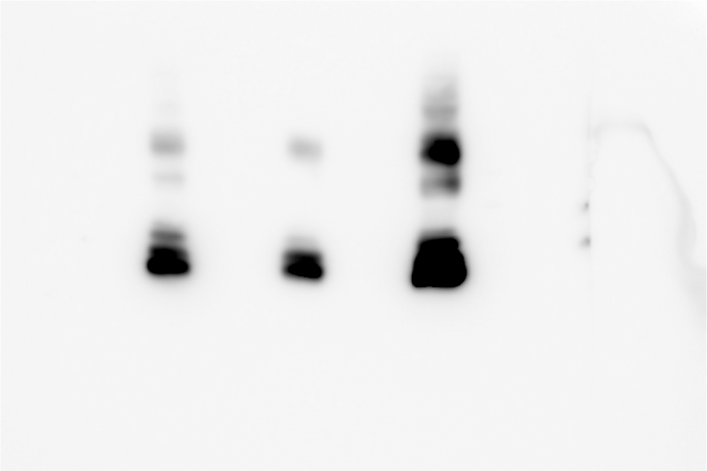

C

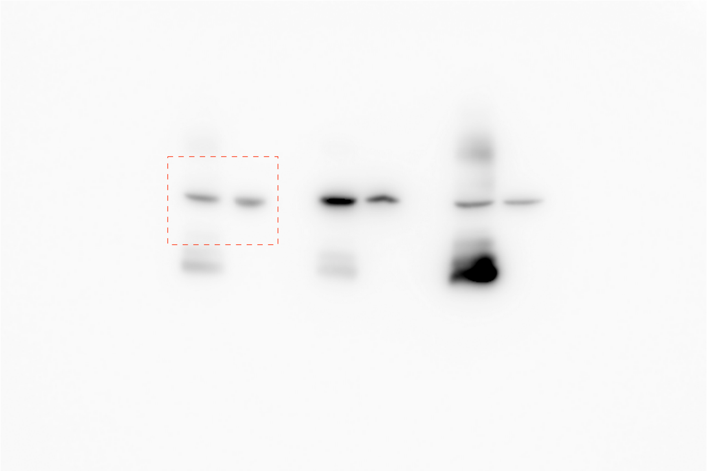

Supplemental Figure S6. The original blots for Supplemental Figure S1C
(**A**) The original blot used for Supplemental Figure S1C using anti-AQP4 C-terminal domain followed by HRP-labeled anti-rabbit IgG. The cropped area produced using Canvas X Draw ver. 7.0.4 (Canvas GFX Inc., Boston, MA) is indicated by red dotted line. (**B**) Longer exposure of the same membrane presented in (**A**) is shown in order to make membrane edge visible. (**C**) The same membrane used in (**A**) was re-probed with HRP-labeled anti-actin antibody. The cropped area is indicated by red dotted line.

Supplemental Table 1. The list of primers used for determination of the structure of AQP4 transcripts.

ID	*Sequence*
S 1	5′-ATGAGAGCTGCACTCCGGCCAGGGAAGGCATGAG-3′
S 2	5′-AGTGACGGAGCTGCAGCGAGGCGGTGGGGTAAGTG-3′
S 3	5′-GGACCTCCCTGCAGCAGAGAGAGCATCATGGTGGC-3′
S 4	5′-CTACCTGTGGACATGGTCCTCATCTCCCTCTGC-3′
S 5	5′-GTGGAAGGCATGGAAATCTCTGTGTGGGGTTGGTG-3′
S 6	5′-GAAACCTCACTGCTGGCCATGGGCTCCTGGTG-3′
AS 1	5′-CACCAGGAGCCCATGGCCAGCAGTGAGGTTTC-3′
AS 2	5′-ACATACTCGTAAAGTGCACCTGCCAGCACAGC-3′

Supplemental Table 2. Sizes of RT-PCR products amplified from each predicted transcript.

Transcripts	*Primer Sets*					
	S1-AS1	S2-AS1	S3-AS1	S4-AS1	S5-AS2	S6-AS2
(i)	515 bp	482 bp	447 bp	291 bp		298 bp
(ii)			447 bp	291 bp		298 bp
(iii)	1553 bp	1520 bp	1484 bp		458 bp	298 bp
(iv)			1484 bp		458 bp	298 bp
(v)	100 bp					298 bp
